# Supplementary material for: The Impact of Scholastic Factors on Physical Activity Levels during the COVID-19 Lockdown: A Prospective Study on Adolescents from Bosnia and Herzegovina
Source: Children (Basel). 2021 Oct 1;8(10):877. doi: 10.3390/children8100877 (PMC8534587; doi:10.3390/children8100877)
Supplement: Supplementary file 1 [file children-08-00877-s001.zip › children-1399883-supplementary.pdf]

Supplementary Materials

Table S1. Characteristics of the participants (F – frequencies, % - percentages).

	**F**	**%**
Gender		
Male	284	53.1
Female	246	45.9
Missing	5	0.9
Grade point average		
Excellent	205	38.7
Very good	200	38.3
Good	104	19.4
Sufficient	7	1.31
Insufficient	7	3.18
Missing	2	0.37
School absences		
< 5 hours	229	42.8
5-10 hours	208	38.9
11-20 hours	80	15.0
> 20 hours	18	3.36
Missing	0	0
Unexcused absences		
< 5 hours	380	71.0
6-10 hours	98	18.3
11-15 hours	24	4.5
16-20 hours	15	2.8
> 20 hours	18	3.4
Missing	0	0
Behavioral grade		
Excellent	456	85.2
Very good	52	9.8
Proper	16	3.0
Poor	11	2.1
Missing	0	0
Sport participation		
Never been involved	145	27.1
< 1 year	107	20
2-5 years	154	28.8
> 5 years	129	24.1
Missing	0	0
Parental education		
Elementary	40	7.5
High school	388	75.5
College degree	60	11.2
University degree	47	8.8
Missing	0	0
Parental conflict		
Never	213	39.8
Rarely	204	38.1
From time to time	105	19.6
Regularly	13	2.4
